# Eliminated routine postorthotopic liver transplant antibiotics in uncomplicated patients leads to equivalent safety outcomes

**DOI:** 10.1017/ash.2021.239

**Published:** 2022-01-24

**Authors:** Jesica Yau, Jillian Dann, Jennifer Geyston, Heather Cox Hall, Shawn Pelletier, Costi D. Sifri

**Affiliations:** 1 Department of Pharmacy Services, University of Virginia Health System, Charlottesville, Virginia; 2 Division of Transplantation Surgery, Department of Surgery, University of Virginia Health System, Charlottesville, Virginia; 3 Division of Infectious Diseases & International Health, Department of Medicine, University of Virginia Health System, Charlottesville, Virginia

## Abstract

**Objective:**

The purpose of this retrospective study was to evaluate safety and efficacy end points of a postoperative antibiotic prophylaxis protocol in liver transplant (LT) patients, which was revised to limit antibiotic use.

**Methods:**

In the routine antibiotics group (RA), patients routinely received prophylactic antibiotics for around 3 days postoperatively for a variety of rationales, versus the limited antibiotics group (LA), in which patients received antibiotics for the treatment of secondary peritonitis. Patients were included if they were 18 or older and underwent liver transplant between January 2016 and September 2019. In total, 216 patients remained after exclusion: 118 patients in the RA group and 98 patients in the LA group.

**Results:**

We detected a significant difference in the primary end point of postoperative antibiotic days of therapy. The median days of therapy was 2 for the RA group and 0 for the LA group (*P* < 0.005). Significantly fewer patients received only intraoperative antibiotics in the RA group versus the LA group: 42 (35.6%) versus 76 (73.5%) respectively (*P* < .005). There was no significant difference in secondary or safety outcomes, including surgical site infections.

**Conclusions:**

This study provides evidence that limiting the duration of prophylactic antibiotics postoperatively and treating most patients with only intraoperative antibiotics is safe.

Surgery in solid organ transplant (SOT) recipients is associated with higher rates of surgical site infection (SSI) than non-SOT recipients when similar clean or clean-contaminated operations are compared.^1–6^ In liver transplantation, SSIs have a reported incidence from 4% to 45%.^4–12^ This increased rate of infections is thought to be due at least in part to the technical complexity of SOT surgeries, the poor state of health of most recipients, and the immunosuppressive agents given to prevent rejection. SSIs following transplantation have also been shown to increase hospital stay, readmission rates, and cost of hospitalization.^13,14^ Furthermore, SSIs are associated with an increase in mortality and graft failure following transplantation.^4,14^ Due to the significance of SSIs in transplant, various strategies have been employed to reduce the rate of these infections, including perioperative antibiotic prophylaxis.

Perioperative antibiotic prophylaxis is standard of care for SOT surgery; however, the duration of postoperative antibiotics and specific antibiotics used varies between different transplant centers and the organ(s) transplanted. A survey of European transplant centers published in 2009 assessed the then-current practices for antibiotic prophylaxis in orthotopic liver transplant (OLT) recipients and found no consensus on the specific agents used.^15^ These researchers reported a range of duration of use from 2 to 7 days, with a median of 3 days. An even wider range of prophylaxis duration can be found in the literature down to as little as a single dose.^16^

The American Society of Health-System Pharmacists, Infectious Diseases Society of American, Surgical Infection Society, and Society for Healthcare Epidemiology of America (ASHP/IDSA/SIS/SHEA) collaborative guidelines for antibiotic prophylaxis in surgery include recommendations for agent selection and duration of perioperative antimicrobial prophylaxis. The guideline specifies piperacillin–tazobactam or cefotaxime with ampicillin for 24 hours or less.^17^ The guidelines from the American Society of Transplantation (AST) Infectious Diseases Community of Practice mirror those of the aforementioned recommending either piperacillin–tazobactam or a third-generation cephalosporin plus ampicillin for up to 24 hours.^18^ It has been suggested that antimicrobial prophylaxis is unnecessary beyond 24–48 hours because there is no evidence of benefit of prolonged prophylaxis; however, whether or not there is a benefit in SOT has not been firmly established.^17,18^

In June 2018, as a part of a systemwide antimicrobial stewardship effort at the University of Virginia Medical Center, a revised perioperative protocol was implemented for liver transplant recipients. Prior studies have examined risk factors for intra-abdominal infections (IAIs) and SSIs, and the following risk factors have been identified: liver transplant, prolonged operative time, decreased donor liver-to-recipient body mass ratio, pretransplant ascites, non-IAI surgical complications (eg, biliary leak, stenosis, or reoperation for bile leak, bleeding, or vascular reconstruction), hepaticojejunostomy, and the need for hemodialysis after transplant.^4,22^ The protocol implemented in June 2018 integrated some of these risk factors to determine antibiotic duration. This practice change provided an opportunity to study the effects of a protocol that was designed to limit postoperative antibiotics. We evaluated the effect of stewardship efforts to eliminate postoperative antibiotics on antibiotic exposure and safety following OLT.

## Methods

### Study design and population

This retrospective cohort study included consecutive adult liver transplant recipients at the UVA Medical Center from January 2016 through September 2019. This study was approved by the Institutional Review Board of the University of Virginia. Data were extracted and recorded from the electronic medical record using both automated report functions and manual audits, and data analysis and preparations were carried out in SPSS version 24 software (IBM, Armonk, NY). The following exclusion criteria were applied: multiorgan recipients, patients with a prior transplantation, patients receiving targeted antibiotics through the transplant period (including those receiving treatments for positive donor cultures), patients who died within 24 hours of transplantation, and those who received a transplant in June 2018 (ie, the transition period during which the new protocol was implemented). Patients who underwent a repeat transplantation had their first transplant included, but the repeat transplantation was excluded.

Cohorts were defined as the routine antibiotics group (RA), which included patients undergoing OLT between January 2016 to May 2018 and the limited antibiotics group (LA), which included patients undergoing OLT between July 2018 and September 2019. The primary end point was days of antibiotic therapy (DOT) for antibiotics given after transplant surgery. Secondary end points included in-hospital mortality, mortality, graft loss, death censored graft survival, SSI, *Clostridioides difficile* infection (CDI), infections, length of stay, rate of readmission, and indication for readmission. Follow-up for secondary end points was 30 days from transplantation except for readmission rates, which had a follow-up of 30 days from the date of the discharge from the index stay.

### Immunosuppression

The center’s immunosuppression protocol remained constant over the study period. Patients received basiliximab, methylprednisolone, and mycophenolate mofetil for induction according to our institutional protocol. Maintenance immunosuppression included tacrolimus with a goal trough of 8–10 ng/mL for the first 3 months, mycophenolate mofetil, and a steroid taper. Induction for patients with primary diagnosis of autoimmune hepatitis included the same immunosuppressive regimen above with the option to substitute rabbit antithymocyte globulin for basiliximab at provider discretion.

### Perioperative antibiotic regimens

The antibiotic regimen of choice for perioperative and postoperative antibiotics for the RA and LA group was either piperacillin–tazobactam or, for penicillin-allergic patients, aztreonam, vancomycin, and metronidazole. In the RA cohort, patients received extended-duration antibiotics due to an indication conferring higher risk of infection. Such recognized indications were bowel perforation during surgery, open abdomen after surgery, complicated bile leak, Roux-en-y hepaticojejunostomy, or reoperation in the immediate postoperative period. The duration of such postoperative antibiotics was based on provider preference. For patients without one of the previously mentioned indications, postoperative antibiotics were generally not recommended in standard care plans; however, this practice was not firmly reviewed or audited.

In the LA group, postoperative antibiotics were limited to the treatment of secondary peritonitis. Secondary peritonitis included 4 indications: bowel perforation during transplant, open abdomen after surgery, a complicated bile leak, or a return to the operating room (OR) for anything other than a simple washout or second look. Recipients who had further repair, reconstruction, anastomosis, thrombectomy or other surgical instrumentation were still considered to be indicated for postoperative antibiotics. Patients with these indications were to receive 7–10 days of postoperative antibiotics. Institutionally, it was felt that the patients meeting these high-risk indications deserved prophylaxis durations similar to that of a treatment regimen. Patients without a higher risk for SSI indication receive no additional antibiotics beyond those administered intraoperatively.

### Definitions

Days of antibiotic therapy (DOT) was defined by the Centers for Disease Control and Prevention (CDC) National Healthcare Safety Network (NHSN) days of therapy, which states a day of therapy is defined by any amount of a specific antimicrobial agent administered in a calendar day to a particular patient as documented in the electronic medication administration record summed in aggregate. DOT included prophylactic antibiotics initiated postoperatively. Intraoperative antibiotics only was defined as receiving antibiotics prior to closure of the skin. Receiving targeted antibiotics through the transplant period was defined as antibiotics that had been initiated before the transplant operation and were continued postoperatively. These targeted antibiotics were not considered in the tally for DOT because postoperative antibiotics related to the transplantation were the focus of this study. Antibiotics received for treatment of infections, such as pneumonia or urinary tract infections developed during the hospital stay, were not counted toward DOT. SSIs were defined by the NHSN criteria, and superficial incisional, deep incisional, and organ-space SSIs were summed in aggregate.^23^ Patients with suspected infections in the abdomen or potential infections that did not meet CDC criteria for organ-space SSIs were characterized as having secondary peritonitis. Urinary tract infections (UTIs), bloodstream infections (BSIs), and pneumonias were also defined using CDC criteria. Spontaneous bacterial peritonitis (SBP) was defined by the consensus document published in 2000 by the International Ascites Club.^25^ CDI was defined as a positive polymerase chain reaction (PCR) result in addition to clinical symptoms. Renal function was calculated using the Modification of Diet in Renal Disease Study (MDRD) equation, and values were collected within 1 day prior to OLT surgery.

### Statistical analysis

The primary end point, median DOT, was evaluated using Mann-Whitney *U* analysis because we anticipated that DOT would not be normally distributed. Categorical baseline characteristics and secondary end points were analyzed using χ^2^ analysis. The Student *t* test was used to analyze means of ordinal data. A *P* value ≤.05 was considered statistically significant.

## Results

In total, 325 OLT surgeries were performed during the study period. After exclusion criteria were applied, 216 patients remained: 118 patients in the RA group and 98 patients in the LA group. Patients were excluded (n = 109) for the following reasons: multiorgan transplantation (n = 26), prior transplantation (n = 13), age <18 years old (n = 19), transplantation in June 2018 (n = 11), and targeted antibiotics through transplantation (n = 40). No significant differences were found in the baseline characteristics between the RA and LA groups (Table 1).

**Table 1.** Baseline Characteristics

Variable	RA Group (n = 118)^a^	LA Group (n = 98)^a^	*P* Value
Age, mean y [±SD]	53.7 [10.9]	55.5 [10.8]	.221
Sex, male	70 (59)	64 (65)	.367
BMI, kg/m^2^ [±SD]	29.6 [5.9]	29.9 [5.5]	.733
**Race or ethnicity**			.099
White	103 (87)	86 (88)	
African American	10 (8)	7 (7)	
American Indian	1 (0.8)	1 (1)	
Asian	2 (1.7)	2 (2)	
Other	2 (1.7)	2 (2)	
MELD score [±SD)	19.9 [8.0]	18.7 [8.1]	.268
History of SBP 30 days prior to transplant	5 (4)	1 (1)	.152
eGFR, mL/min/1.73 m^2^	76.3	74.7	.690
Roux-en-y hepaticojejunostomy	9 (8)	5 (5)	.453
Living donor OLT	13 (11)	7 (7)	.328
Return to OR	18 (15)	14 (14)	.842
Induction immunosuppression			.667
Basiliximab	116 (98.3)	98 (100)	
Anti-thymocyte globulin	2 (1.7)	0 (0)	

Note. BMI, body mass index; MELD, model for end-stage liver disease; SBP, spontaneous bacterial peritonitis; OLT, orthotopic liver transplant; OR, operating room.

a All values expressed as mean [± SD] or no (%) unless otherwise noted.

**Fig. 1.**
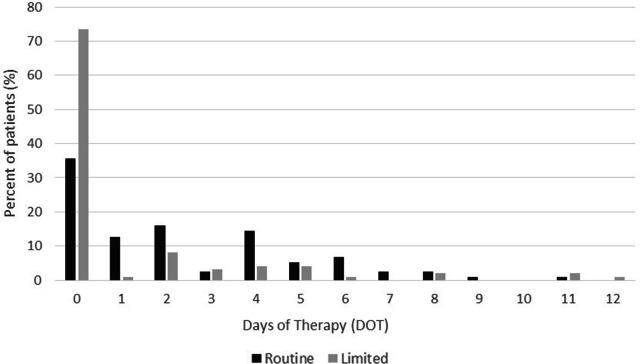
Days of antibiotic therapy (DOT), as defined by CDC NHSN criteria, in Routine (RA) and Limited (LA) groups. Median DOT were lower in the LA group (*P* < .005).

We detected a significant difference in the primary end point of postoperative antibiotic DOT: the median DOT was 2 days for the RA and 0 days for the LA groups (*P* < .005). There was also a significant difference in the number of patients who received intraoperative antibiotics only: 42 (35.6%) and 76 (73.5%) in the RA and LA groups, respectively (*P* < .005) (Fig. 1). The median DOT for patients who received antibiotics postoperatively was 4 days for both the RA and LA groups.

The results for the secondary end points are displayed in Table 2. SSI occurred in 8 patients in the RA group and 5 in the LA group. When SSIs were divided among those who received prophylactic postoperative antibiotics and those who did not, there was no significant difference: 8 (8.2%) of 98 versus 5 (4.2%) of 118 (*P* = .227). For SSIs, 8 (76%) of 13 occurred during the initial transplant admission. When CDIs were divided among those who received prophylactic postoperative antibiotics versus those who did not, there was a significant difference between the groups: 9 (9.2%) of 98 versus 3 (2.5%) of 118 (*P* = .034). A single infection with a multidrug-resistant organism occurred within 30 days after transplantation: 1 patient in the RA group developed a UTI due to vancomycin-resistant *Enterococcus faecium*.

**Table 2.** Secondary End Points

Variable	RA Group (n = 118)^a^	LA Group (n = 98)^a^	*P* Value
In-hospital mortality	3 (2.5)	2 (2)	.807
Mortality at 30 d	3 (2.5)	2 (2)	.807
Graft loss at 30 d	6 (5)	2 (2)	.238
Death censored graft survival at 30 d	97	99	.268
Length of stay, [±SD]	10.6 [7]	12.7 [21]	.308
SSI at 30 d	8 (6.8)	5 (5.0)	.506
CDI at 30 d	8 (6.7)	4 (4.1)	.381
Any infection at 30 d	41 (35)	22 (22)	.212
UTI	6 (5)	4 (4)	
SSI	8 (7)	5 (5)	
Bacteremia	5 (4)	1 (1)	
CDI	9 (8)	4 (4)	
Secondary peritonitis	11 (9)	6 (6)	
Cellulitis	1 (1)	0 (0)	
Dental abscess	1 (1)	0 (0)	
Empyema	0 (0)	1 (1)	
Pneumonia	0 (0)	1 (1)	
Readmission at 30 d from discharge	40 (33)	23 (23)	.093

Note. SSI, surgical site infection; CDI, *Clostridioides difficile* infection; UTI, urinary tract infection.

a All values expressed as mean [± SD] or no. (%) unless otherwise noted.

The overall rates of adherence to the protocol in the RA and LA groups, respectively, were 54% and 70%. Adherence was defined as having received postoperative antibiotics when indicated and not receiving postoperative antibiotics when not indicated. For patients who received antibiotics according to protocol, the median DOT for the RA and LA groups were 5 days (mean, 5.3) and 5 days (mean, 5.8). For patients who received antibiotics outside of recognized indications, some reasons listed included recent fevers, history of spontaneous bacterial peritonitis, breaks in sterility in the OR, mesh in abdomen, pancytopenia, and leukocytosis. However, most patients who received postoperative antibiotics outside the institutionally recognized indications had no indication for continued antibiotic prophylaxis. In fact, 30 patients in the RA group and 6 in the LA group had no indication listed or discovered in the notes for continued prophylactic antibiotics.

Subgroup analyses were performed comparing patients in both groups who received antibiotics without a listed indication versus patients who received no postoperative prophylactic antibiotics. This analysis was performed to attempt to determine whether there was some unforeseen risk for infections in the patients who received antibiotics without a listed indication. We detected no significant differences in age, BMI, MELD, race, and sex between the 2 groups. There was no significant difference in SSIs between the group who received antibiotics without a listed indication and the group who received no postoperative prophylactic antibiotics (1 of 36 and 5 of 118, respectively; *P* = .692).

## Discussion

The purpose of this study was to evaluate efficacy and safety end points of a postoperative antibiotic protocol in OLT patients, which was designed to limit postoperative antibiotics to those with defined risks or indications for antibiotics. This study provides evidence that limiting the duration of prophylactic antibiotics postoperatively and prescribing patients intraoperative only antibiotics is safe in relatively low-risk patients. The LA group had double the percentage of patients who received no prophylactic postoperative antibiotics, despite no increased rate of SSI or infection overall.

These results support the recommendations by the AST and ASHP/IDSA/SIS/SHEA guidelines regarding postoperative antibiotics in liver transplant. Most liver transplant recipients received <24 hours of postoperative antibiotics per this protocol and experienced no change in safety outcomes. Additionally, piperacillin–tazobactam was the first-line agent in this study for intraoperative and postoperative antibiotics, and it is also the first-line agent in both guidelines. Not all patients received therapy strictly per national and international guidelines, however; the median DOT for patients who received postoperative antibiotics in both cohorts was 4 days.

Berry et al^19^ conducted a randomized controlled trial at the same center as our study, examining intraoperative antibiotic prophylaxis versus extended postoperative antibiotics in liver transplant recipients. These researchers found no significant difference in SSIs or nosocomial infections between groups, suggesting that OLT recipients may be adequately protected by intraoperative antibiotics alone. Additionally, studies of renal transplantation have examined the effects of single-dose verses multidose regimens of prophylactic antibiotics and have found similar rates of SSI.^20,21^

Although the protocol in this study did remove 2 indications for prolonged postoperative antibiotics that clinicians treated for, the decreased indications did not fully account for the considerable increase in patients who received no postoperative antibiotic prophylaxis. Adherence to the protocol for low-risk patients accounted for some of the change. Subgroup analysis comparing patients in both groups who received antibiotics for no listed indication versus patients who received no antibiotics revealed no significant difference in SSIs in demographically similar groups, which suggests that the patients who received antibiotics out of the norms or out of protocol may not have benefited from the additional prophylactic antibiotic use. Nevertheless, deviation from protocol for valid provider concerns and clinical judgment should not be universally admonished.

Notably, the rate of SSI in this study was lower than that of a prior prospective trial with similar inclusion and exclusion criteria. Our interpretation of the SSI criteria could have differed from those of other studies, which could have contributed to the lower rate; however, a host of other factors such as patient morbidity and surgeon technique could contribute as well. When comparing these results to the results of Berry et al,^19^ it is unclear what led to the decreased rate of infections. The 30-day SSI rates were 19% for short antibiotics and 27% for extended in the research by Berry et al versus 6.8% and 5% in this study. This study also mirrors the work of Berry et al in that we defined SSIs by the CDC criteria and excluded those under 18 years of age, those with a previous liver transplant, and those requiring antibiotic treatment at the time of transplant.^23^ The only additional exclusion criteria included patients who underwent transplantation in June 2018, the washout period, and patients who died within 24 hours of transplantation. As such, what led to the lower SSI rates remains somewhat unclear, but our results are similar to those of a recent abstract by Hamel et al, which found 30-day SSI rates of 4% and 12% in their liver transplant recipients, respectively.^24^

This study had several limitations. The retrospective design inherently limits the ability to accurately identify certain safety outcomes and other data points due to differences in charting between healthcare workers. Additionally, because patients with prior transplants, multiorgan transplants, targeted antibiotics through transplantation, and the relatively low mean native MELD scores for liver transplant recipients in these cohorts (average score,19.3), these results should not necessarily be generalized to all liver transplant recipients. In fact, prior liver or kidney transplantation has been identified as a risk factor for SSI.^11^ The specific timing of SSI was also not recorded, which limits some of the interpretation of the data for SSIs. This study does provide a decent representation of the bulk of transplant patients because two-thirds of all liver transplantations at our center and during our study period were included in our results.

Given that there was no significant difference in rates of infection in the RA versus LA groups, we propose that a risk stratification strategy could be a beneficial way to determine postoperative antibiotics and reduce antibiotic exposure. Taken collectively, the results of this study and those from other studies support the guidelines recommendations for <24 hours of antibiotic use after transplant. Furthermore, they suggest that intraoperative antibiotics alone without postoperative antibiotics may be safe for uncomplicated liver transplant surgery. However, it remains unknown whether a blanket intraoperative antibiotics only strategy versus a risk-based or indication-based strategy to determine who receives prolonged postoperative antibiotics is superior in terms of SSIs and other safety outcomes. Prospective trials comparing this risk stratification strategy versus intraoperative only would be helpful in determining which strategy leads to superior efficacy and safety outcomes in liver transplant recipients.
